# Antituberculosis Therapy and Gut Microbiota: Review of Potential Host Microbiota Directed-Therapies

**DOI:** 10.3389/fcimb.2021.673100

**Published:** 2021-12-07

**Authors:** Dramane Diallo, Anou M. Somboro, Seydou Diabate, Bacar Baya, Amadou Kone, Yeya S. Sarro, Bourahima Kone, Bassirou Diarra, Souleymane Diallo, Mahamadou Diakite, Seydou Doumbia, Yacouba Toloba, Robert L. Murphy, Mamoudou Maiga

**Affiliations:** ^1^ University Clinical Research Center (UCRC) of the University of Sciences, Techniques and Technologies of Bamako (USTTB), Bamako, Mali; ^2^ School of Laboratory Medicine and Medical Sciences, University of KwaZulu‐Natal, Durban, South Africa; ^3^ Institute for Global Health, Northwestern University, Chicago, IL, United States

**Keywords:** tuberculosis, gut microbiota, TB treatment, host directed-therapies, dysbiosis

## Abstract

Tuberculosis (TB) remains a major public health concern with millions of deaths every year. The overlap with HIV infections, long treatment duration, and the emergence of drug resistance are significant obstacles to the control of the disease. Indeed, the standard first-line regimen TB treatment takes at least six months and even longer for the second-line therapy, resulting in relapses, drug resistance and re-infections. Many recent reports have also shown prolonged and significant damage of the gut microbial community (dysbiosis) from anti-TB drugs that can detrimentally persist several months after the cessation of treatment and could lead to the impairment of the immune response, and thus re-infections and drug resistance. A proposed strategy for shortening the treatment duration is thus to apply corrective measures to the dysbiosis for a faster bacterial clearance and a better treatment outcome. In this review, we will study the role of the gut microbiota in both TB infection and treatment, and its potential link with treatment duration. We will also discuss, the new concept of “Host Microbiota Directed-Therapies (HMDT)” as a potential adjunctive strategy to improve the treatment effectiveness, reduce its duration and or prevent relapses. These strategies include the use of probiotics, prebiotics, gut microbiota transfer, and other strategies. Application of this innovative solution could lead to HMDT as an adjunctive tool to shorten TB treatment, which will have enormous public health impacts for the End TB Strategy worldwide.

## Introduction

Tuberculosis (TB) is the most deadliest single infectious disease driving it at the top of public health priorities, mostly in developing countries, where it is endemic (Arnold et al., 2015; Namasivayam et al., 2018; Namasivayam et al., 2019; Eribo et al., 2020). The *Mycobacterium tuberculosis* (MTB) is the pathogen agent responsible of this disease. It can affect the lung (pulmonary TB) or others body organs (extra pulmonary TB) (Hong et al., 2016). According to the World Health Organization (WHO), about 10 million TB cases and 1.5 million TB deaths were estimated in 2018 with approximately 484 000 cases of drug resistant TB (Global Tuberculosis Report, 2019). About 37% of the world’s population is estimated to be infected with MTB but just 5 to 10% are at risk to develop an active tuberculosis (Luo et al., 2017; Kaipilyawar and Salgame, 2019). In general, the management of TB patients still remain a challenge for medical scientists, particularly patients infected with Human Immunodeficiency Virus (HIV) are more susceptible to TB related death (Luo et al., 2017; Gupta et al., 2018). Furthermore a standard treatment for TB take at least six (6) months for susceptible TB with a combination of four antibiotics (anti-tuberculosis treatments) which are: Isoniazid (INH), rifampicin (RIF), pyrazinamide (PZA), and ethambutol (EMB) (Namasivayam et al., 2017). Despite an efficiency rate of 90% by these anti-tuberculosis treatments (ATT), previously infected and treated TB patients are at 13 times higher risk to develop another episode of tuberculosis (relapses and re-infections) than the general population (Millet et al., 2013). The treatment with this regimen is not without consequences and the main problem is the emerging of resistant to strains (mainly Multi Drug Resistant Tuberculosis, MDR-TB) in which case, we need to extend the regimen and a longer period treatment is required (9-20 months) (Hong et al., 2016; Sood et al., 2018). The impacts of the ATT in the immune system are not profoundly investigated, some studies showed a persistent dysbiosis in the human gut microbiota for a long term after treatment cessation (Wipperman et al., 2017; Hu et al., 2019; Namasivayam et al., 2020). This community of the gut microorganisms help the human immunity in maintaining health status, through the production of various metabolites that influence the immunity (Thomas et al., 2017; Somboro et al., 2021). Metabolites derived from microbial metabolism and microbe-facilitated modulation of host- or dietary-derived metabolites such as bile acids and Short Chain Fatty Acids (SCFAs) are key elements of the crosstalk between the host and its microbial population. Studies have focused on investigating the metabolic capacity of the gut microbiota, with evidence for impact on immune functions at distant sites, including the lung *via* the gut-lung-axis (Brown et al., 2017). Brown et al. demonstrated that the microbiota enhances respiratory defenses *via* granulocyte-macrophage colony-stimulating factor (GM-CSF) signaling, which stimulates pathogen elimination and clearance by alveolar macrophages through extracellular signal-regulated kinase signaling (Brown et al., 2017). Increased pulmonary GM-CSF production in response to infection is primed by the microbiota through interleukin-17A. Changes in the diversity of microbial community can cause a disorder in the host defense and make it susceptible to several diseases (Sood et al., 2018) such as inflammatory bowel disease, multiple sclerosis, diabetes (type I and type II), allergies, asthma, autism and cancer (Lloyd-Price et al., 2016). The gut microbiota is known to have an immunomodulatory role by providing signals to promote the maturation of immune cells and the normal development of immune functions (Hu et al., 2019). This role makes, the gut microbiota critical therapeutic target for TB. Any major perturbation of the microbiota (*e.g.*, antibiotics, genetic, gender, age) has to be accounted as influencer of the disease and treatment outcome, one way or the other (Namasivayam et al., 2018). In human the factors that are linked to gut microbiota dysbiosis are meanly exemplified by the dietary regimen, where the type of nutriments that are consumed can impact on the gut microbial population (Leeming et al., 2019). It is known that the western diet is mostly high in fat and this is associated to a decrease of bacterial community in the human gut thus, leading to the degradation of the intestinal mucus, reduced level of SCFAs, with higher risk of pathogen to invade the gut. In contrast the Mediterranean diet is higher in fiber than fat, therefore, the fiber-based diet provides beneficial bacterial species and nutrients to the gut microbiota and reactivate the production of SCFAs (Schweitzer et al., 2019). Exposure to pathogens is another factor that lead to gut dysbiosis. The colonization of the gastro-intestinal tract by pathogenic organisms induces inflammation that perturbate the gut microbiota, with consequences, the imbalance in microbe’s composition and function. Age, physical activities, psychological stress and/or anxiety, drug usage, smoking of tobacco and alcohol consumption are all potential factor intervening in the gut dysbiosis (Wen and Duffy, 2017). More investigations are needed to study and understand the link between these perturbations in the microbial community and the involved biological processes including but not limited, to the immune resistance and the development of drug resistance. In this review, we are seeking to focus on understanding the effects of anti-tuberculosis treatment on gut microbiota by summing up the recent results and discuss possible host targeted gut microbiota as a way to change and improve disease and treatment outcomes. We will call this new concept, “Host Microbiota-Directed Therapy for TB”.

## Collection Strategy

In this review, the literature search was conducted using PubMed and Google Scholar as libraries and without date specification. We searched references primarily with the following terms: “Tuberculosis treatment”, “gut microbiome”, “gut microbiota” “dysbiosis” and “Host-Directed therapies”. Articles were then collected and reviewed.

## Antituberculosis Treatment

TB treatment regimen can vary from six months for newly infected cases to nine months for multi-drug resistant tuberculosis cases (MDR-TB). Newly TB diagnosed patients are treated with a fixed dose combination of two months of rifampicin (R), isoniazid (H), pyrazinamide (Z), and ethambutol (E) and four months of (R) and (H) (2RHZE/4RH; Category 1) while retreatment patients receives two months of (R), (H), (Z), (E), and streptomycin (S), followed by 1 month with the same combination but without (S) and five months of (R), (H), and (E) (2RHZES/1RHZE/5RHE; Category 2) (Diarra et al., 2016). The extensively long term of treatment can subdue patients under adverse reactions which are related to various factors, and the principal determinants of such reactions are the dose and time of day at which the medication is administered, and age and nutritional status of the patient, together with its health conditions, such as alcoholism, impaired liver and kidney functions, and HIV co-infection. These discomforts due to medication can be either minor or major adverse effects. The minor advert effects include nausea, vomiting, abdominal and epigastric pain, arthritis, arthralgia, cutaneous pruritus, peripheral neuropathy, headache, and behavioral changes (anxiety, insomnia, low libido, and euphoria). The major adverse effects are mostly expressed through psychosis, vertigo, exanthema, and hepatotoxicity (Arbex et al., 2010). It is known that the treatment of TB ends up with various inconveniences, particularly the occurrence of TB drug resistance, the dysfunction of gut microbial organisms affecting the host health.

## Human Microbiome and Healthy Status

The definition of the term “microbiome” was first given by Whipps et al. in 1988, where they described the microbiome as a combination of the words “micro” and “biome”, referring to a characteristic microbial community in a reasonably well-defined habitat that exhibits distinct physiochemical properties as their theater of activity (Whipps et al., 1988). Thereafter many other definitions of the term “microbiome” were given in different reports such as the one described by Thomas et al. and Sood et al.., defining the microbiome as “the total genome of complete commensal, symbiotic and pathogenic community of microorganisms shared in each area of our body” (Thomas et al., 2017; Sood et al., 2018). More recently, Berg eet al. combined the categories of definitions and defined the microbiome as “ecological community of commensal, symbiotic, and pathogenic microorganisms that literally share our body space” (Berg et al., 2020), while microbiota refers to the microbes that live in a specific location (Hong et al., 2016). The human microbiome is influenced by several factors (e.g. life style, gender, environmental changes) (Conlon and Bird, 2015). Furthermore, the dietary type has been shown to be highly implicated in the microbiome structural composition (Adithya et al., 2021). The type of diet modulates the abundance of altered bacteria in the gut microbiota at various stage of life. Individuals with high-fiber diets have their gut populated with beneficial microbes, which could exert health benefits, while the gut microbiome composition of those with high-fat and high-sugar diets are negatively altered (Adithya et al., 2021). The diet patterns have been correlated to the host intestinal microbiota interaction with many metabolic processes, and is responsible in part in protecting or predisposing multiple metabolic, immunological, neoplastic, and functional diseases (Schiumerini et al., 2018; Adithya et al., 2021).

Composed by trillion microbes, human microbiota is implicated in several biological processes, in the pathogenesis and the progression of diseases (infectious diseases, liver diseases, gastrointestinal cancers, respiratory diseases, mental, metabolic and autoimmune diseases) (Wang et al., 2017). For instance, a Disruption of the human microbiota is convincedly associated with the pathogenesis of *Clostridium difficile* infection (Young, 2017). Another study has related the increase in the Firmicutes and decrease in the Bacteroidetes in the gut microbiota of patients and animal models with obesity (Conlon and Bird, 2015). Further, several studies have recently reported the interaction between host microbiota, immune system and the oncogenesis process (Conlon and Bird, 2015; Thomas et al., 2017). A new study from Oxford University showed a significance relation between reduced gut microbiome compositions and participants behavioral traits, stress and anxiety while larger social network people have been found to have more diverse microbiome (Johnson, 2020).

## Impact of Antituberculosis Treatment on the Gut Microbiome

**Figure 1:**
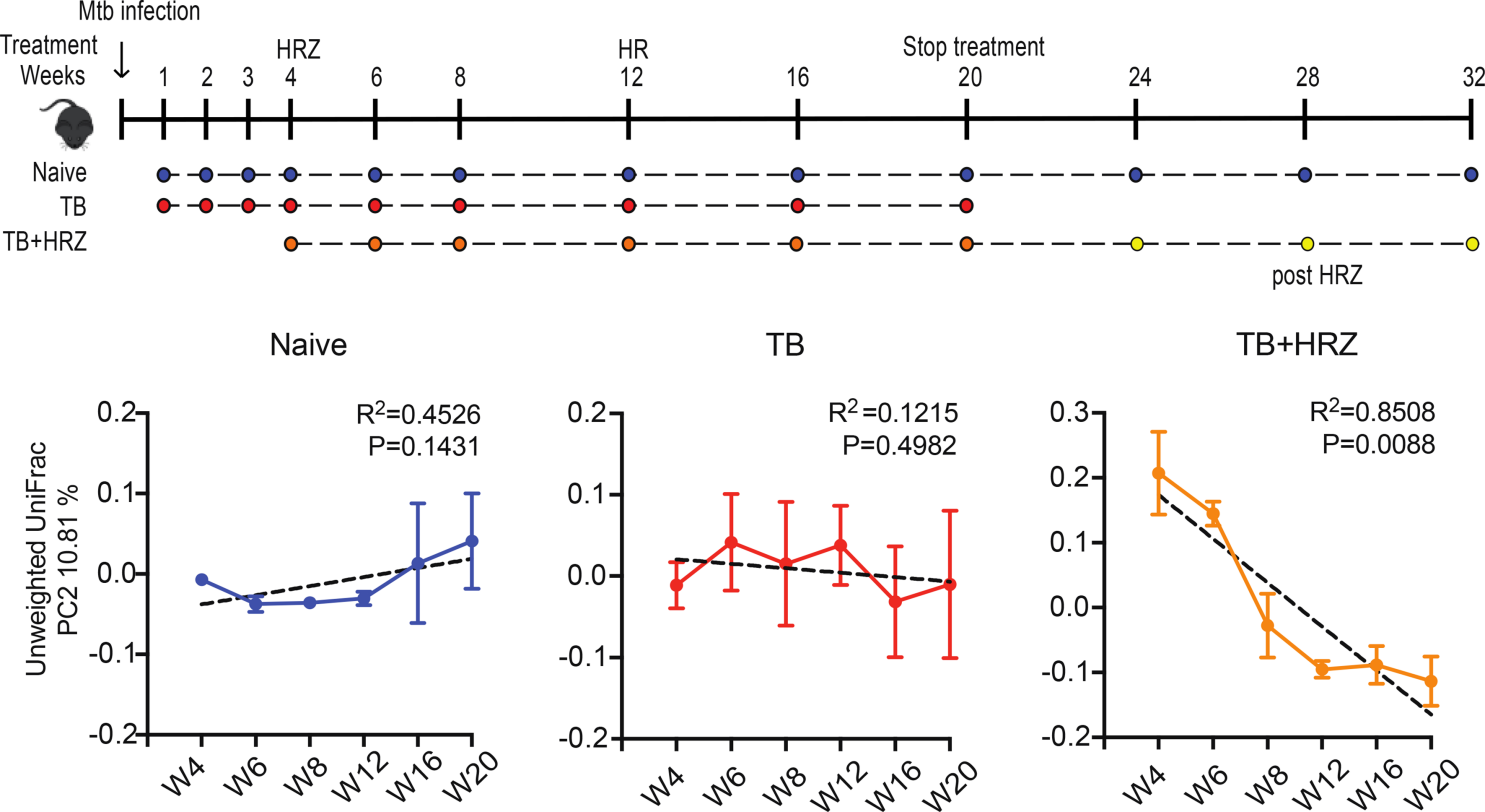
In the mouse model, TB first-line drugs induce a distinct and long lasting dysbiosis. X-axis = weeks of treatment; Y-axis = bacterial diversity; TB = TB infected, HRZ = isoniazid+rifampin+pyrazinamide treated (Namasivayam et al., 2017).

Although, ten million patients receive TB drugs annually worldwide, little is currently known about the impacts and consequences of the antituberculous drugs on gut microbiota during and after drug treatment. Using a mouse model, Namasivayam and colleagues showed that the first-line TB therapy, made of two months of isoniazid (H), rifampin (R), pyrazinamide (Z) and four months of HR (2HRZ/4RH), profoudly changes the gut microbiome (**Figure 1**), with a long lasting dysbiosis after treatment cessation (Namasivayam et al., 2018). In 2013, Millet and colleagues found that patients who have previously TB-infected and treated are at 13 times higher risk of MTB reinfection than the general population (Millet et al., 2013). This could be the consequence of dysbiosis occurring during TB treatment, indeed *Pseudomonas* is more abundant in sputa of patients who fail treatment compared with cured or new patients, and the ratio of *Pseudomonas/Mycobacterium* in treatment failure patients is higher than in new TB patients (Wu et al., 2013). In the mouse model with first line ATT, the order Clostridiales decreased during treatment while the family Porphyromonadaceae increased at the end of the treatment. In a clinical study, it was found that ATT impact significantly certain bacterial populations, such as decreasing the family Enterobacteriaceae (phylum Proteobacteria) (Namasivayam et al., 2017; Namasivayam et al., 2020). A recent study on the effects of intestinal microbiome on INH efficacy using mice model showed changes into the gut microbiome population and the abolishment of innate immune response against MTB during INH therapy by a downregulation in the expression of MHC-II and CD86 which diminished the antigen presentation ability and activation of myeloid DCs in lung. Moreover a reduction of the expression of innate receptors (*TLR2*, *Mincle* and *Nod2*) caused by microbiota disruption was observed in MTB infected mice under INH therapy (Negi et al., 2020). Those findings raise the possibility that the composition of the microbiota may influence treatment outcome and future resistance against the disease.

## Microbiota Products and Their Impacts on Our Immune System

Host immune system is regulated in part by microbes through production of metabolites. This is evidenced by studies reporting that, diverse microbial metabolites intensely regulate the immune system *via* host receptors and other target molecules (Rooks and Garrett, 2016; Levy et al., 2017; Haase et al., 2018; Kim, 2018). To respond to changes in health conditions, nutrition status, and immune regulation, the metabolites together with receptors form an extensive array of signals, thus contributing into nutrient harvest from diet, regulating host metabolism and the immune system. Microbiota products considered here as microbial metabolites serve to promote host immunity and tolerance to control infection without developing inflammatory diseases (Belkaid and Hand, 2014). It is known that bacteria produce short-chain fatty acids (SCFAs) resulting in carbohydrate fermentation in the colon and express carbohydrate-active enzymes (e.g. glycoside hydrolases, polysaccharide lyases), to process long carbohydrate fibres into simple sugars (Levy et al., 2016). Accumulation of certain microbial metabolites in colon, notably SCFAs, causes reduction of pH, regulation of microbial function and composition, fulfils nutritional needs, and conditions the immune system. Human microbiota produces several molecules to influences the host innate immune system (Namasivayam et al., 2018). Sharon Perry and team reported that patients with latent TB infection (LTBI) and *Helicobacter pylori* possess in peripheral blood mononuclear cells, higher TB antigen-induced IFN-c responses and a more Th-1 like cytokine profile, than of those who were *H. pylori* seronegative (Perry et al., 2010). Metabolomics studies focusing on small molecule metabolites that impact the host metabolome and their biochemical functions, have shown promises for studying human gut microbiota interactions and investigating host microbiota as therapeutic options (Chen et al., 2019).

## Pharmacomicrobiomics

Orally drug administration pass through the gastrointestinal tract until the intestinal tract, where it facing the second immune defense (microbiota) (Weersma et al., 2020). According to the composition of intestinal microbiota, intake drug structure is changed, which impact its pharmacological parameters, such as its bioavailability, bioactivity and its efficacy (Scher et al., 2020; Weersma et al., 2020). Controversially, drug can impact the microenvironment (dysbiosis) by decreasing some microorganisms’ population (Weersma et al., 2020). Pharmacomicrobiomics help to well understanding this complex interaction between drug and gut microbiota and open the way to an individual personalized medicines (Doestzada et al., 2018). Gopalakrishnan and collaborators reported in 2018, the modulation of the response to anti-programmed cell death 1 protein (PD-1) immunotherapy in melanoma patients (Gopalakrishnan et al., 2018). A panel of antibiotics has also been investigated by Cussotto et al. to understand the influence of microbiota in these drugs absorption, however, this study did not include anti-tuberculosis drugs (Cussotto et al., 2019). An *in vivo* mouse model to study the influence of gut microbiota dysbiosis on isoniazid (INH) efficiency against *M. tuberculosis*, was conducted by Liu et al. and they found that a declined abundance of *Lactobacillus, Bifidobacterium*, and *Campylobacter* caused by antibiotic pre-treatment is susceptible impairing the immune response to isoniazid treatment in *M. tuberculosis* clearance. Moreover the impairment of the intestinal innate defense and immunity stemmed from microbiota changes during INH therapy (Negi et al., 2020; Liu et al., 2021). More detailed studies are required to better understand the gut microbiota-anti-TB drugs interaction.

## Host Microbiota Directed Therapy

Antibiotics have been introduced into human therapy for many years and save countless lives and remain essential in clinical practice (Butler et al., 2013), however, their usage profoundly impacted human associated microbial communities (Langdon et al., 2016). The use of antibiotics often works with help from the immune system by immunomodulation, nevertheless, it can perturb the ecological system by killing symbiotic bacterial communities thus, creating a rare opportunity for a pathogenic population, whose growth usually is suppressed by the symbiotic bacterial to become a dominant species in the community. The role of a microbiota-directed therapy would then be in this situation, to adequately target the detrimental population of bacteria to shift it to a mutualistic bacterial community. Therefore, strategies such as probiotics, prebiotics, gut transplantation therapies could be an ideal assets to efficiently control diseases.

### Probiotic Therapies

Probiotics are products used as treatment option, that are part of microbiota-targeted class of therapies, where their therapeutic properties are intimately connected to ecological principles. They are usually components of bacteria or live microbial food supplements with effects that are beneficial to human health (Salminen et al., 1998). The Food and Agricultural Organization of the United Nations and the World Health Organization FAO/WHO classified probiotics as “*live microorganisms that, when administered in adequate amounts, confer a health benefit on the host*” (Hill et al., 2014). Clinicians commonly prescribe probiotics, often when requested by the patient himself and also as an antidote to the potential side effects of antibiotics. Most of these products claim to promote and reconstitute the microbiota composition in the body and they target different part of the human body and diverse diseases.

- *Probiotics targeting the gut*: Many probiotic products possess the potentiality to balance the gut microbiota and to prevent the replication of pathogenic bacteria thus, conferring health. Probiotics play essential role in the restoration of the gut microbiota composition and establish beneficial functions to the microbial communities, that will result in the improvement or prevention of gut inflammation and other intestinal or systemic disease phenotypes. *Lactobacillus rhamnosus* was the first probiotic to gain attention in this field and has shown beneficial effects on intestinal immunity (Pandey et al., 2015). It enhances the integrity of the intestinal barrier, which will in turn help to maintain the immune response, decrease the translocation of bacterial population across the intestinal mucosa (Blackwood et al., 2017). Researchers currently recommend to explore whether the association of TB drug with probiotics targeting the gut will improve treatment response and outcome (Eribo et al., 2020)
- *Probiotics targeting skin, oral, vaginal, and upper respiratory tract*: In other parts of human body where microbial communities exist other than the gut, probiotics have been evaluated in order to explore their efficacy to treat and/or prevent infectious diseases (Bustamante et al., 2020). The effects of probiotics on the treatment of infections occurring in the female urogenital tract, the skin, and respiratory tract have been investigated and the evidence has demonstrated that the use of probiotics by mothers during pregnancy and lactation can prevent or reduce the presence of eczema in their children (Bustamante et al., 2020). Recurrent urinary tract infections can as well be prevented by probiotics consumption (Tariq et al., 2017). The use of mutualistic bacterial products can reduce respiratory tract infections and impact into their duration and severity (Bustamante et al., 2020).
- *Probiotics targeting other aspect of human diseases*: Probiotic products act through inhibition of the onset of allergic diseases (Toh et al., 2012), they prevent and treat ischemic heart syndromes (Antony and de Leon, 2018), and used as therapeutic option for rhinosinusitis (Schwartz et al., 2016), cure for epilepsy (He et al., 2017a) and can improve life of patients with inflammatory bowel disease (Wei et al., 2015). However, other health benefits require additional studies, such as cancer prevention, treatment of acute diarrheal diseases, and anticarcinogenic properties (Nagpal et al., 2012; Kechagia et al., 2013).

### Prebiotics Therapies

The term prebiotics refers to “a substrate that is selectively utilized by host microorganisms conferring a health benefit” (Gibson et al., 2017). The definition expands the concept of prebiotics to possibly include non-carbohydrate substances (e.g. fatty acids, phytochemicals and phenolics), topical applications other than the GIT, and diverse categories other than food. The most commonly used prebiotics are the fermentable oligosaccharides such as, inulin, galacto-oligosaccharides, fructo-oligosaccharides, and lactulose (Swanson et al., 2020). Mohamad Miqdady et al. recently reviewed the clinical evidence of prebiotics and discussed their role in gut immunity, infections, allergy, inflammation, and functional gastrointestinal disorders (Miqdady et al., 2020). Based on the extremely low risk of serious adverse effects, the ease delivery, the high potential in influencing the gut microbiota composition and function plus their clinical benefits the resumed that prebiotics seems to be promising therapeutics option.

All the above mentioned therapeutic options can be implemented to TB in order to prevent and treat in an efficient manner the deadly infection, and now a day researchers are focus in such strategies to combat diseases, particularly TB.

## Potential Host Microbiota Directed-Therapies for Tuberculosis

The increasing attraction of microbiome as target for potential therapeutic options, is due to extensive role in different disease outcomes. The majority of current microbiome-related therapies are directed to the prokaryotic group of the microbiome, commonly termed probiotics that affect the microbial population of the gut *via* exogenous administration of live microbes (Zeng et al., 2019). Alternatively prebiotics are also investigated, so, instead of administrating live bacteria to patients, products (prebiotics) that are used in the intention of regulating the microbiome population or function will be employed with effects that are beneficial to the recipients. Thus far, there is no FDA-approved microbiome-directed therapy for TB, however, since 2013 it has been included in the FDA guidelines, the use of faecal microbiota transplantation (FMT) as treatment guidelines for relapsing *Clostridium difficile*. Studies are now been directed in stepping-up treatment strategy of FMT for diverse pathologies with or without associating conventional drugs (Fischer et al., 2015; Wei et al., 2015; He et al., 2017a; He et al., 2017b; Zeng et al., 2019).

In the last decades, various microorganisms are used as probiotic, prebiotic or symbiotic to treat and/or prevent certain number of diseases, such as diarrhea, cancer, irritable bowel syndrome, inflammatory bowel disorder (Ulcerative colitis, Crohn’s disease, Pouchitis), Cardiovascular diseases etc. It was demonstrated that probiotics comprising of cocktail of bacterial species and yeast (*Lactobacillus acidophilus, Lactobacillus rhamnosus, Lactobacillus delbruckii, Lactobacillus. fermentum, Saccharomyces boulardii*) are effective in reducing the incidence of antibiotic-induced diarrhea. It was also shown that the yeast *Saccharomyces boulardii have* better effectiveness on diarrhea caused by bacterial infections, while the *Lactobacillus GG* possess more efficacy against viral and idiopathic diarrhea. To prevent traveller’s diarrhea, *Bifidobacteria*, *Lactobacilli, Streptococci, Enterococci* are used as prophylactic strategy (D’Souza et al., 2002; Varankovich et al., 2015).

It is known that people with unhealthy population of gut bacteria are more predisposed to develop colon cancer (Zhang et al., 2015). In 2010, Gianotti L. et al. administered a strain of *Lactobacillus johnsonii* to colorectal cancer patients, that was able to adhere to the colonic mucosa, leading to the reduction of gut pathogens concentration and the modulation of the local immunity (Gianotti et al., 2010). Demers M. et al, in 2014, after a double-blinded and controlled trial, unveiled the beneficial role of *Lactobacillus acidophilus* and *Bifidobacterium longum* in decreasing radiation-induced diarrhea, when administered to cancer patients under pelvic radiation therapy (Demers et al., 2014). Theodoropoulos Ge et al., in 2016, used a combination of prebiotics and probiotics (synbiotics) in patients subjected to colorectal cancer resection to decrease the risk of developing post-operatory irritable bowel syndrome (Theodoropoulos et al., 2016). In the same year, Consoli ML et al. conducted, another study which involved patients with colon-resected colorectal cancer and found that the strain *Saccaromices bulardii* efficiently downregulated the expression of pro-inflammatory cytokines (Consoli et al., 2016). A recent clinical study revealed the significant benefit of using a synbiotic mixture of probiotics and prebiotics, through the reduction of postoperative infection rates in patients with colorectal cancer (Flesch et al., 2017).

An association of lactic acid bacteria and propionic acid bacteria was found to be highly efficient for treatment of inflammatory diseases (Devi et al., 2013). Due to the bactericidal property of lactic acid bacteria against *Mycobacterium tuberculosis*, this can be a promising research topic in the development of new and efficient probiotics for complex treatment of tuberculosis. The challenge toward developing host microbiota directed-therapies for TB is determining the cause-effect relationships and designing the microbiota-based therapies that will be able to achieve predictable outcomes on the microbial community and host health. Many lung microbiota-targeted treatments have been proposed for many respiratory diseases (e.g., Ashman, chronic obstructive pulmonary disease) (Chung, 2017; Vaughan et al., 2019) that could be use in TB context. The spray of phage inhaler directly to the lung, is promising but requires further research to establish the best formulation to use (e.g., powder freeze drying, liquid aerosols, nebulization), its true additive clinical value and safety to patients (Leung et al., 2016). Probiotics and prebiotics are microbiota-based interventions promising approaches to tackle TB infection, thus, further studies are required for in-depth understanding and characterization of their effects.

Host-directed therapies are options that are destined to improve the success of tuberculosis treatment through immunomodulation/immune enhancement in order to eliminate the residual bacteria that are less sensitive to the antibiotics. On the other hand, the immune enhancement is being considered acting in synergy with the anti-tuberculosis treatment regimens in order to improve long-term outcomes and promote recovery from the infection (Young et al., 2020).

**Figure 2:**
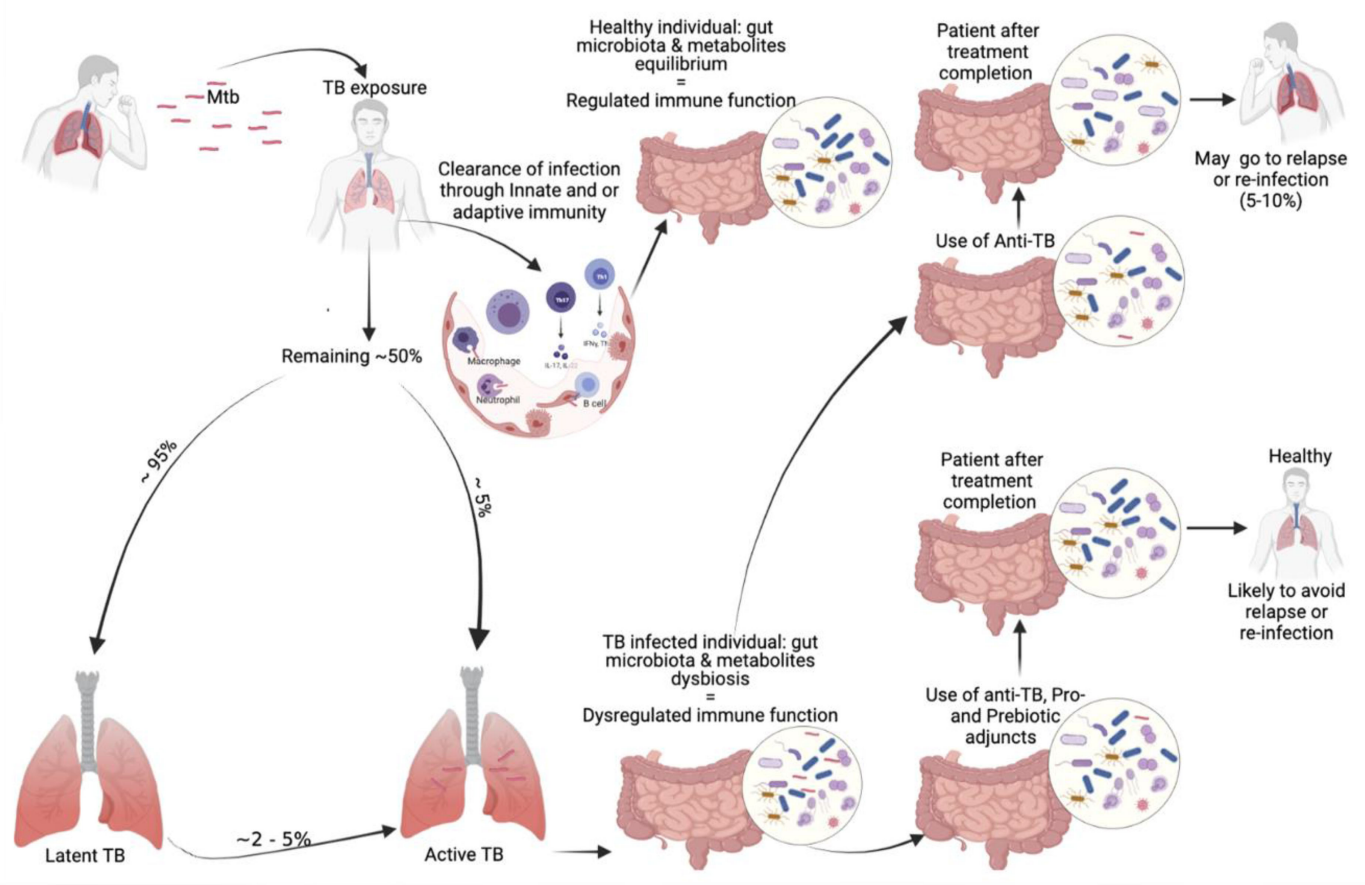
Our proposed involvement of the microbiota in TB infection and possible microbiota-targeted-adjuvants during tuberculosis treatment.

Tuberculosis infection occurs through fine aerosol particles containing the infectious agent. The newly infected individual may clear the infection by the help of the innate and/or adaptive immune defensive processes in which the human gut microbiota does contribute significantly. The majority of newly infected individuals may progress to latent TB infection (LTBI) and about 5% will progress to active TB as illustrated in **Figure 2**. Individuals with LTBI may fully clear their infection or progress to active TB disease depending on their immune condition, which is influenced by the microbiota and many other factors. The host microbiota contributes to the early protection against the colonization of human lung by MTB. The gut microbiota and metabolites equilibrium leading to immune function homeostasis help in preventing individuals to progress to LTBI or TB disease (Lin and Zhang, 2017). Once in active TB state, a dysbiosis is observed in the host gut microbial population. The gut microbiota dysbiosis is also observed during anti-tuberculosis therapy, that will persist many months after treatment completion. Those treated with TB antibiotics have higher chance to be re-infected than the general population (Millet et al., 2013) (Hermans et al., 2021). Host microbiota directed therapies can be a promising approach as adjuvants to TB treatment by recovering the immune protective gut microbial populations that were eliminated by TB drugs (**Figure 2**). The immune enhancement acts in synergy with the TB drugs in order to improve long-term outcomes (Young et al., 2020).

## Conclusion

Host microbiota-based therapies are promising and can serve as potential supportive treatment options for several diseases including tuberculosis for which the scientific community is struggling to find new option. Especially, the fact that TB drugs dramatically damage the gut microbiome, demonstrates the urgent need for alternative treatment strategies. Interventions to the gut or the local respiratory microbiota are possible paths that need to be investigated further to determine their clinical utilities in supporting the TB antibiotics’ treatment and prevent re-infections in post-treatments.

## Author Contributions

All authors listed have made a substantial, direct, and intellectual contribution to the work, and approved it for publication.

## Funding

This publication was supported by the HBNU Consortium, Fogarty International Center and the National Institutes of Health under Award Number D43TW010543, R21AI148033 and the Northwestern Medicine Institute for Global Health Catalyzer Funding.

## Author Disclaimer

The content is solely the responsibility of the authors and does not necessarily represent the official views of the National Institutes of Health.

## Conflict of Interest

The authors declare that the research was conducted in the absence of any commercial or financial relationships that could be construed as a potential conflict of interest.

## Publisher’s Note

All claims expressed in this article are solely those of the authors and do not necessarily represent those of their affiliated organizations, or those of the publisher, the editors and the reviewers. Any product that may be evaluated in this article, or claim that may be made by its manufacturer, is not guaranteed or endorsed by the publisher.
